# Investigating the Effect of Social and Cultural Factors on Drivers in Malaysia: A Naturalistic Driving Study

**DOI:** 10.3390/ijerph182211740

**Published:** 2021-11-09

**Authors:** Ward Ahmed Al-Hussein, Miss Laiha Mat Kiah, Lip Yee Por, Bilal Bahaa Zaidan

**Affiliations:** 1 Department of Computer System and Technology, Faculty of Computer Science and Information Technology, University of Malaya, Kuala Lumpur 50603, Malaysia; wva180034@siswa.um.edu.my (W.A.A.-H.); misslaiha@um.edu.my (M.L.M.K.); 2 Department of Computing, Faculty of Arts, Universiti Pendidikan Sultan Idris, Tanjong Malim 30000, Malaysia; bilalbahaa@fskik.upsi.edu.my

**Keywords:** driving behavior, naturalistic driving study, aggressive driving, speeding, driver performance, the relationship between social/cultural factors and driving, characteristics of young and older drivers

## Abstract

Road accidents are increasing every year in Malaysia, and it is always challenging to collect reliable pre-crash data in the transportation community. Existing studies relied on simulators, police crash reports, questionnaires, and surveys to study Malaysia’s drivers’ behavior. Researchers previously criticized such methods for being biased and unreliable. To fill in the literature gap, this study presents the first naturalistic driving study in Malaysia. Thirty drivers were recruited to drive an instrumented vehicle for 750 km while collecting continuous driving data. The data acquisition system consists of various sensors such as OBDII, lidar, ultrasonic sensors, IMU, and GPS. Irrelevant data were filtered, and experts helped identify safety criteria regarding multiple driving metrics such as maximum acceptable speed limits, safe accelerations, safe decelerations, acceptable distances to vehicles ahead, and safe steering behavior. These thresholds were used to investigate the influence of social and cultural factors on driving in Malaysia. The findings show statistically significant differences between drivers based on gender, age, and cultural background. There are also significant differences in the results for those who drove on weekends rather than weekdays. The study presents several recommendations to various public and governmental sectors to help prevent future accidents and improve traffic safety.

## 1. Introduction

Following cardiovascular diseases and cancer, traffic accidents are the third leading cause of death [1]. Despite the government’s various preventive measures, the number of road accidents in Malaysia is increasing year after year [2]. From 2000 to 2015, the country saw a 93 percent increase in total vehicle accidents, resulting in a 17 percent increase in mortality [3]. Malaysian road users are categorized as the worst in Southeast Asia, with 23.8 deaths per a 100,000 population [4]. In general, three main factors contributed to road accidents: human, vehicle, and road condition. Accidents are caused by the human factor alone in 80–90 percent of cases [4]. According to these statistics, most road accidents are caused by reckless driving, which has been identified as the leading cause of road accidents in most countries.

In the literature, researchers reported that social factors, such as age and gender, impact driving [5,6,7,8,9,10,11]. Researchers in reference [12] identified age, gender, national, and regional differences as impacting drivers’ behavior. Moreover, researchers discovered that driving on weekdays may have a negative impact on safe driving because drivers are under tremendous pressure to get to work [13]. Furthermore, researchers have identified a number of factors that influence driver performance, such as driving experience, level of education, and knowledge, concluding that novice drivers are more likely to underestimate hazards than experienced drivers, who are often more focused [14]. Previous studies also showed a statistically significant relationship between lifestyle dimensions, such as an aggressive driving association with driving for recreational purposes, and a safe driving association with people of a religious background [15].

It is important to investigate the relationship between driving behavior and social/cultural factors because they are determinative to aggressive driving.

It is also becoming increasingly necessary to comprehend the impact of such factors on driving in Malaysia, as cultural differences influence driving in various countries [16]. Another reason is that conclusions in the literature have often been contradictory; therefore, they cannot be generalized. For instance, researchers long believed that male drivers are more likely to be involved in motor-vehicle crashes [17]; however, female drivers are now over-represented in crashes when compared with males [9].

Regarding Malaysia, previous researchers attempted to identify differences between driving in Malaysia and driving in the UK in references [18,19]. Moreover, one study looked at the differences in road safety attitudes and driver behavior between Malaysia and Singapore [20]. Furthermore, researchers determined the characteristics of basic driving skills among older drivers in Malaysia [21]. Those studies, however, had limitations. First, they did not examine the impact of the social/cultural factors on drivers in Malaysia, which raises the following questions:

1. Do social factors, such as gender and age, influence drivers’ behavior in Malaysia?
2. Is driving in Malaysia influenced by one’s cultural background, such as whether one is a local or a foreigner?
3. Is there a difference between driving on a weekday and driving on a weekend in Malaysia?

Another common issue within the studies that aimed at understanding drivers’ behavior in Malaysia is the use of inadequate and biased techniques for data collection such as surveys, questionnaires, simulations, and on-road observations using cameras, which were heavily criticized in the literature [9,10,22,23,24,25,26,27,28,29,30,31,32,33,34,35,36,37,38]. As a result, collecting naturalistic driving data (NDD) using in-vehicle sensors emerged as a crucial data source with high ecological reliability [39,40]. NDD have been widely used to predict the likelihood of various behaviors such as reckless speeding, lane changing, and distracted driving. Based on speeding behaviors extracted from vehicles’ GPS trajectory data, researchers in [41] classified drivers into three categories: restrained, moderate, and belligerent. Researchers also utilized existing datasets to investigate the effects of gender and age on speeding [42]. Moreover, in [43], researchers demonstrated a model that detects lane-changing maneuvers using the SHRP2 dataset. Furthermore, researchers utilized NDD to compare the safety of organized and unorganized carpooling situations in terms of speeding and distractions while driving [44]. In Malaysia, researchers used in-vehicle sensors to collect data [2]. However, those experiments were conducted in a non-naturalistic manner. Two cones were placed on the street by the researchers. They asked drivers to focus on steering through the cones rather than braking, which altered their natural driving style, resulting in the experiments being non-naturalistic. The main objectives of this study are as follows:

1. Develop a reliable and cost-effective data acquisition system (DAS) for gathering driving data in naturalistic experiments. The proposed DAS should be as unobtrusive to the drivers as possible, so that it does not interfere with their natural driving style.
2. Compile a dataset of over 750 km of continuous driving data from 30 drivers across two cities in Malaysia.
3. Investigate the influence of social/cultural factors on driving behavior in Malaysia.

## 2. Methodology

### 2.1. DAS Design and Installation

The proposed DAS consisted of an onboard diagnostics (OBDII) reader, a lidar, two ultrasonic sensors, an inertial measurement unit (IMU), and a standalone global positioning sensor (GPS). The sensors used in the proposed DAS, their location inside the vehicle, and the corresponding recorded data are shown in Table 1.

#### 2.1.1. OBDII

**Figure 1:**
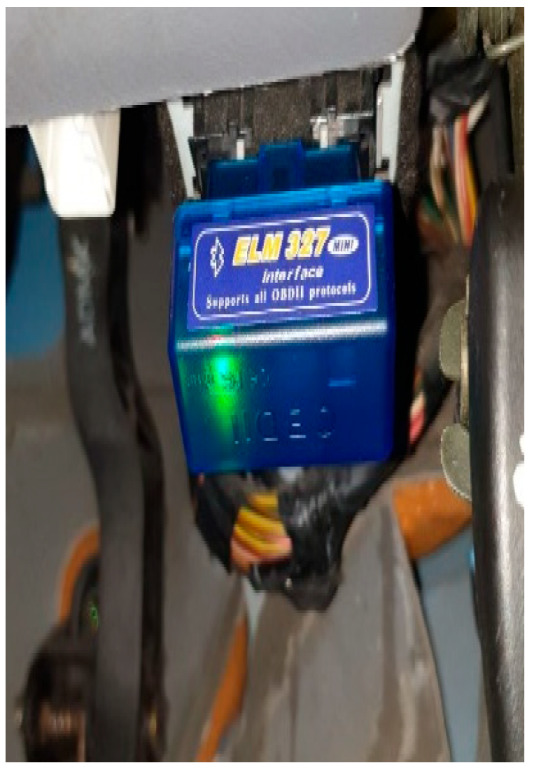
Installation of ELM327 inside the vehicle.

The selected OBDII reader was ELM327. It is cost-efficient, is easy to plug into the vehicle’s OBDII 16-pin connecter, and can send data to a smartphone via Bluetooth in Excel format. The communication between the smartphone and the ELM327 was sent via an application called Torque. Figure 1 shows the ELM327 installed beneath the steering wheel. The smartphone was placed inside the vehicle’s glove compartment.

#### 2.1.2. Lidar

It is critical to choose a lidar that can detect objects accurately. Initially, the plan was to use the lidar model TF03, which can cover distances of up to 100 m. During the installation phase, however, tests revealed low detection rates. The Garmin lidar has a better reputation among sellers; however, it only covers up to 50 m. Other commercially available models cover no more than 22 m at the most, such as TF02, TiM100, TFMini, and RPLIDAR. Table 2 compares the TF03 lidar to the Garmin lidar in a 30 min test drive on a medium-traffic route in the Serdang area.

**Figure 2:**
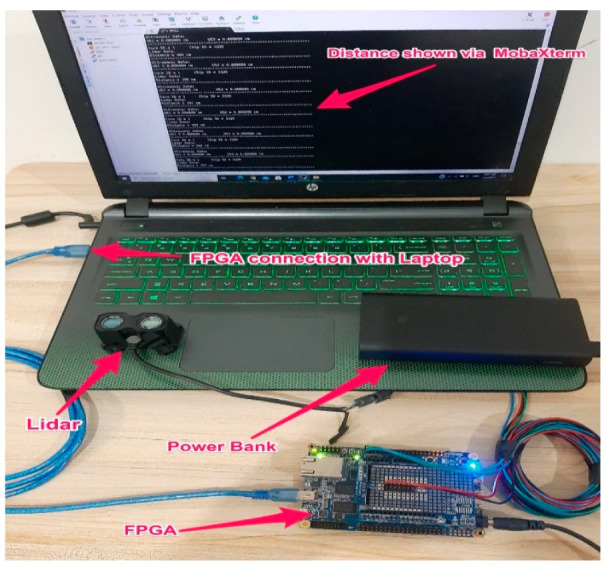
The connection between the laptop, FPGA, and lidar.

Table 2 shows that the Garmin lidar recorded more distance data than the TF03 lidar (73% vs. 32%). Furthermore, the Garmin lidar outperformed the TF03 lidar in terms of detection rate (82% vs. 45%). Therefore, as a result, the Garmin lidar was chosen for the proposed DAS. It was placed on top of the license plate to measure the distances between the experimental vehicle and vehicles in front of it. The lidar was connected via cables to Terasic DE10-Nano, a field-programmable gate array (FPGA), for its flexible and reconfigurable design, high processing capability, and high-speed DDR3 memory. To provide stable electrical power for the lidar, the FPGA was connected to a power bank. MobaXterm, a terminal software installed on a laptop, was used to send commands to the FPGA to start and stop the recording process of distance data. The data were then saved in text format to the FPGA’s secure digital (SD) card. The physical connection between the lidar, FPGA, and laptop is depicted in Figure 2.

**Figure 3:**
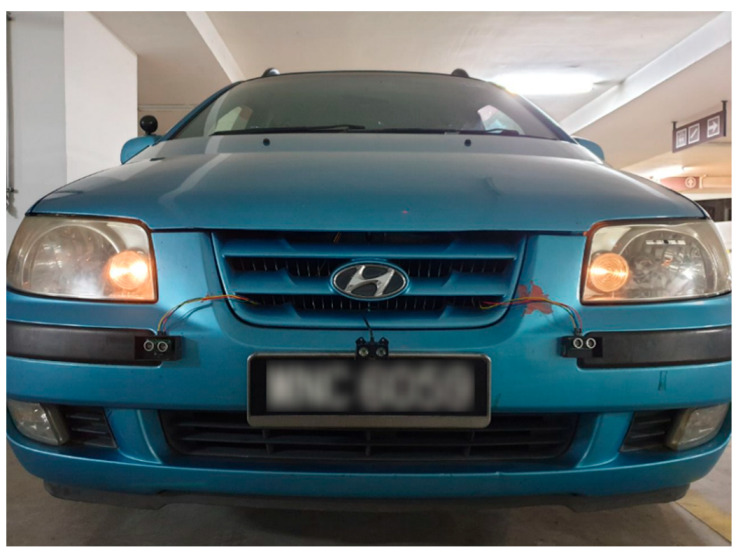
Installation of ultrasonic sensors and Garmin lidar on the vehicle.

**Figure 4:**
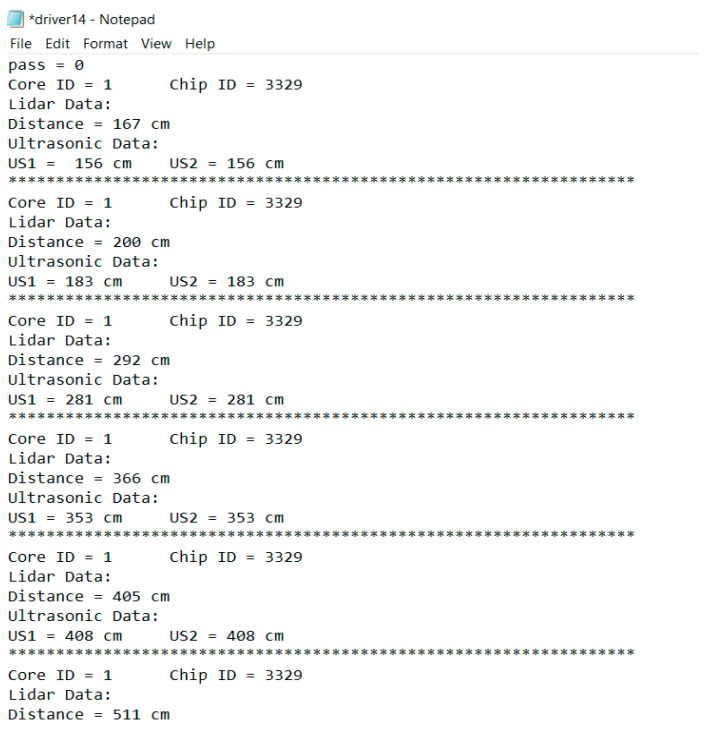
Sample of distance data recorded by FPGA in text format.

It is commonly known that lidars are typically not very accurate in detecting objects over short distances. As a result, ultrasonic sensors were used for short-range detection. Two ultrasonic sensors were placed on the left and right sides of the Garmin lidar. The FPGA was reconfigured and programmed to record distance data from the two ultrasonic and lidar sensors simultaneously. This combination of the Garmin lidar and two ultrasonic sensors increased detection accuracy from 82% to 97–100% in distances up to 50 m. Figure 3 shows the installation of the ultrasonic sensors and the Garmin lidar on the vehicle. Figure 4 shows a sample of distance data recorded by FPGA from the two ultrasonic sensors and the Garmin lidar.

After configuration, the FPGA was placed inside the vehicle trunk and connected to the Garmin lidar and ultrasonic sensors via 12 cables, 6 m long, wrapped in protective sleeves.

#### 2.1.3. IMU

**Figure 5:**
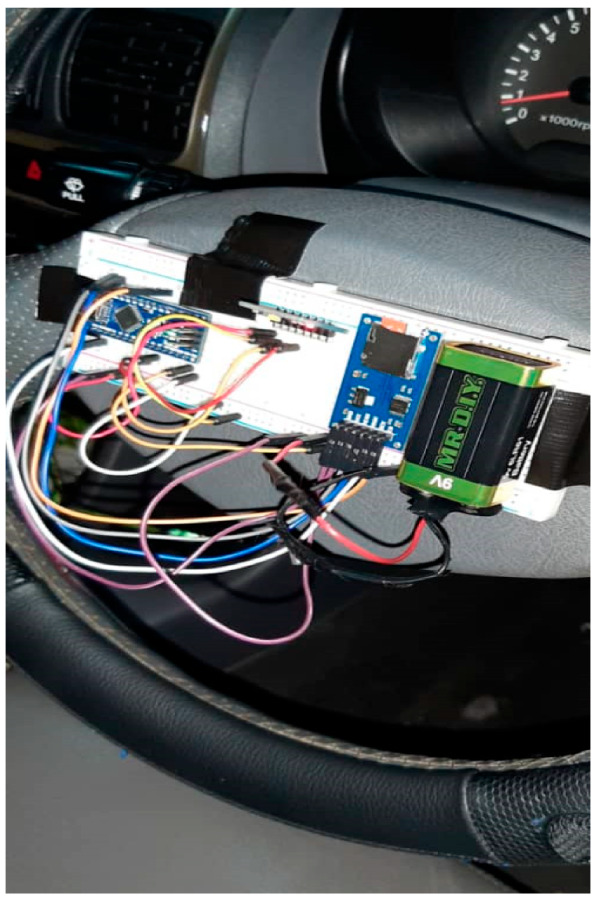
Early IMU design.

The MPU-6050 model was chosen because it is cheap, small-sized, and easy to install and configure. The first design was simple: installing the IMU on the steering wheel along with a microcontroller, a battery, and a SD card. The system worked, but there were two major issues. First, the battery had to be replaced every two hours, rendering the proposed system impractical. Second, placing the IMU directly on the steering wheel has its own set of issues, as drivers may accidentally touch the IMU, causing their natural driving behavior to change and the system to crash. Figure 5 depicts the early design for the IMU system.

The design of a small enough system to be placed inside the steering wheel makes the proposed system non-visible to the drivers. In addition, it eliminates possible biases from the data collection process. As a result, the IMU system of the proposed DAS was distributed into two parts: the sending side and the receiving side.

**Figure 6:**
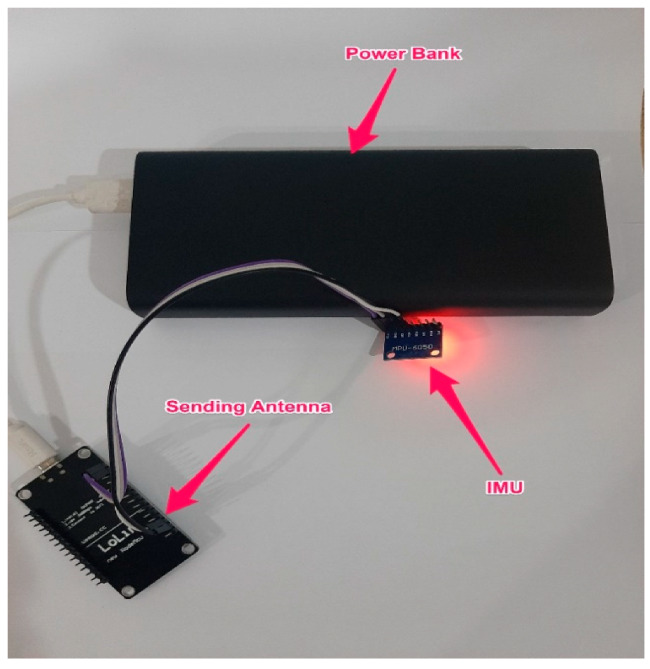
Sending side of the IMU system.

Figure 6 shows the sending side of the IMU system, which is installed inside the vehicle’s steering wheel. On the sending side, the IMU sensor was connected to an antenna that sends recorded steering data to another antenna on the receiving side. The antenna on the sending side was connected to a power bank.

**Figure 7:**
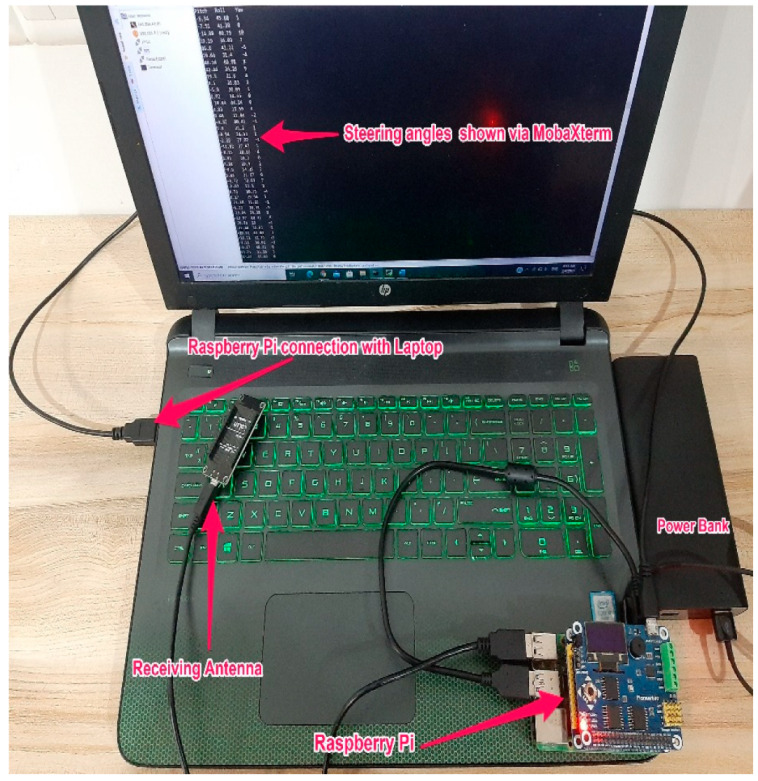
Receiving side of the IMU system.

**Figure 8:**
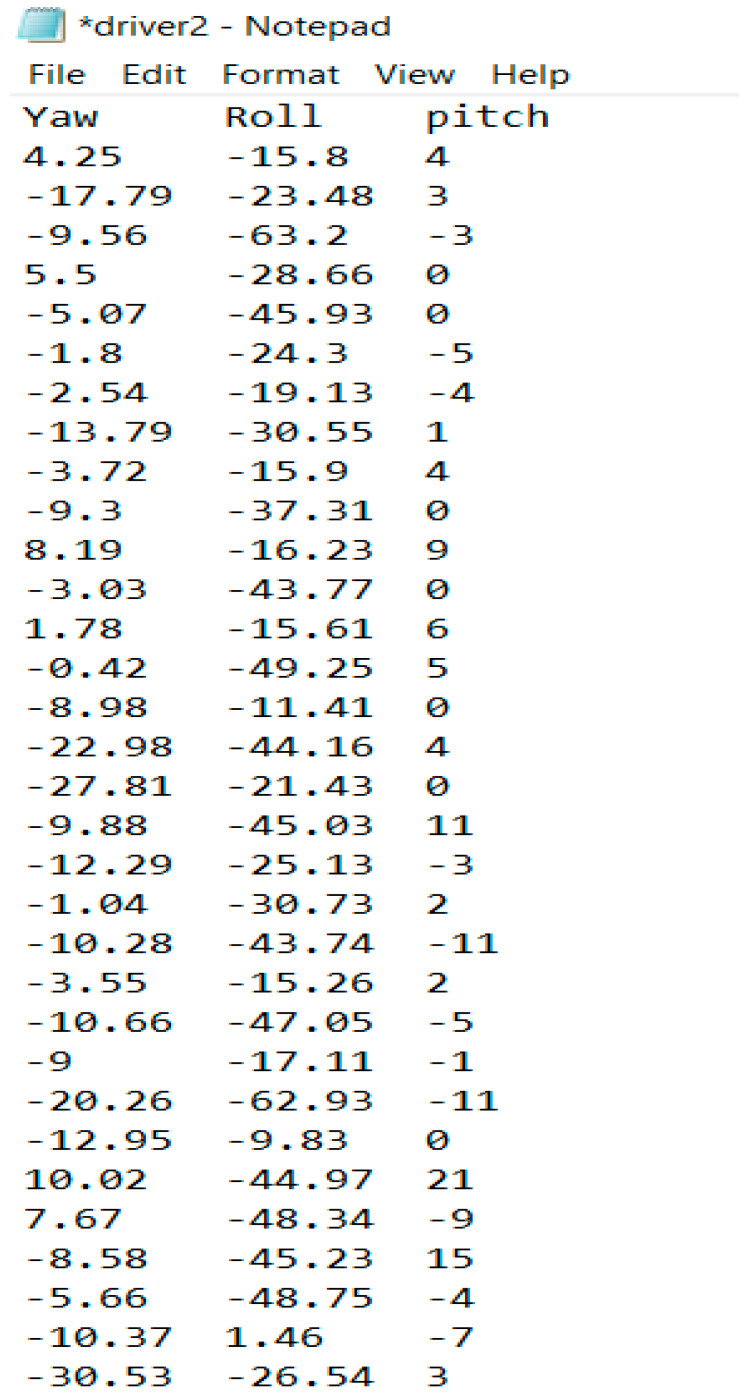
Sample of steering data recorded by raspberry pi in text format.

Figure 7 shows the receiving side of the IMU system, which was placed inside the vehicle trunk. A raspberry pi, an antenna, a power bank, and a laptop comprised the receiving side. The laptop was connected to a raspberry pi board (model Raspberry PI-4B-4G). Using a raspberry pi instead of a standard microcontroller is advantageous because the raspberry pi is a minicomputer that has a faster clock speed, has more RAM, performs multitasking, and consumes less power. The antenna on the receiving side was in charge of delivering the commands from the laptop to the antenna on the sending side and obtaining recorded steering data. The recorded data were saved in text file format to the raspberry pi SD card. Figure 8 shows a sample of steering data in text format.

It is worth noting that the MPU-6050 includes a DMP (Digital Motion Processor) that implements the MotionFusion algorithm, which fuses data from the gyroscope and accelerometer to minimize sensor errors. As discussed previously, on the sending side of the system, the MPU-6050 was placed inside the steering wheel on a flat horizontal surface. As a result, the returning values on the receiving side of the system would represent the driver’s steering wheel rotation 

It is worth mentioning that Jeff Rowberg and I2Cdev libraries [45] provide a collection of classes that support simple interfaces to inter-integrated circuit devices, for researchers interested in calibrating the sensor. The offset values of the gyroscope’s axis in the raw code should be set to zero. The offsets should be adjusted based on the values returned by the code until the code returns 0 for every axis.

**Figure 9:**
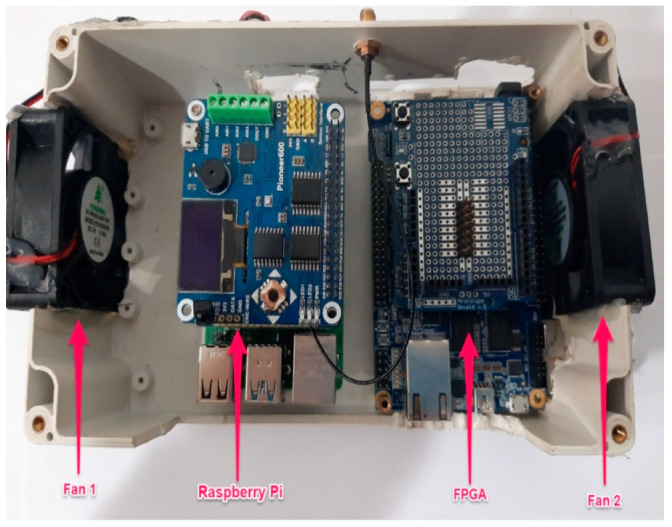
Data collection box.

**Figure 10:**
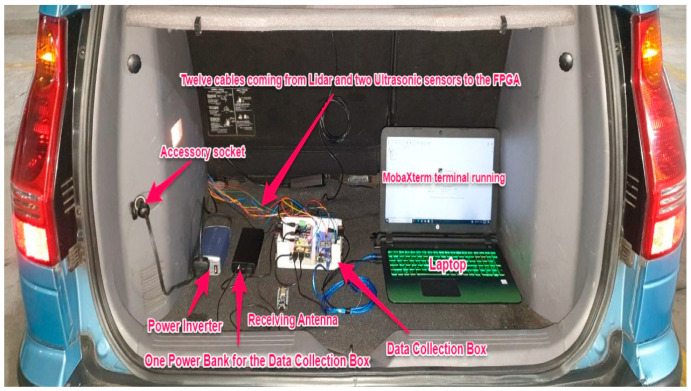
Equipment inside vehicle’s trunk.

A case was designed and built to protect the raspberry pi and the FPGA located inside the vehicle’s trunk. This protective case was named the data collection box. Two fans were installed on the left and right sides of the data collection box to protect the sensitive boards from dust and overheating. In addition, a power inverter was plugged into the vehicle’s accessory socket to prevent the laptop from running out of power during experiments. Figure 9 shows the data collection board, and Figure 10 shows the entire equipment inside the vehicle trunk.

An external GPS device was installed inside the vehicle to ensure that drivers did not deviate from the predetermined route. 

Participating in a driving study may impact drivers’ behavior because sensors and devices may cause distractions or inconveniences to participants, altering their normal driving patterns. Thus, to minimize the possibility of influencing drivers’ behavior, a unique DAS design was proposed, in which most of the chosen sensors and related equipment were well hidden within the vehicle. As previously stated, the OBDII reader was placed on the right side of the vehicle beneath the steering wheel. The smartphone was placed inside the glove compartment. The sending side of the IMU system was placed inside the steering wheel, while the receiving side of the IMU system was placed inside the vehicle’s trunk along with the other equipment, such as the laptop, FPGA, and raspberry pi. Finally, the lidar and the ultrasonic sensors were placed in front on top of the license plate. 

**Figure 11:**
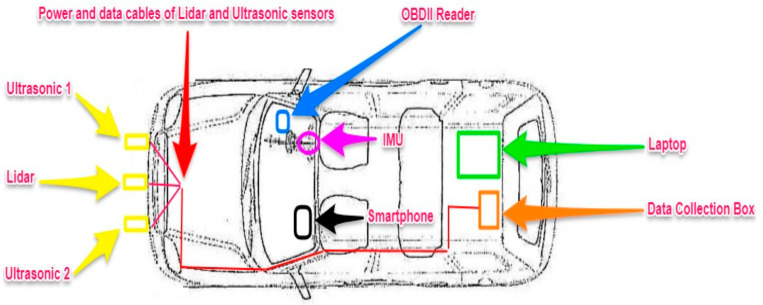
The architecture and design of the proposed DAS.

Furthermore, researchers in [46] advocated for the development of a reliable, cost-efficient DAS. As a result, the proposed DAS had a total cost of only 406 dollars (excluding the laptop and the smartphone). Figure 11 depicts the proposed DAS’s entire architecture and design.

### 2.2. Participants

A total of 30 participants were recruited in this study, 15 of whom were males and 15 were females. There were 15 locals and 15 foreigners. Their mean age was 40.96 years. The youngest participant was 20 years old, and the eldest was 69 years old. Participants were categorized into three groups based on their age (young, middle, and senior). Each group consisted of 10 participants. The young group included participants between the age of 20 and 29. The middle group included participants between the age of 30 and 49. Finally, the senior group included participants between the age of 50 and 69. On average, they have had 22.28 years of driving experience, ranging from 2 years to 51 years.

### 2.3. Test Route 

**Figure 12:**
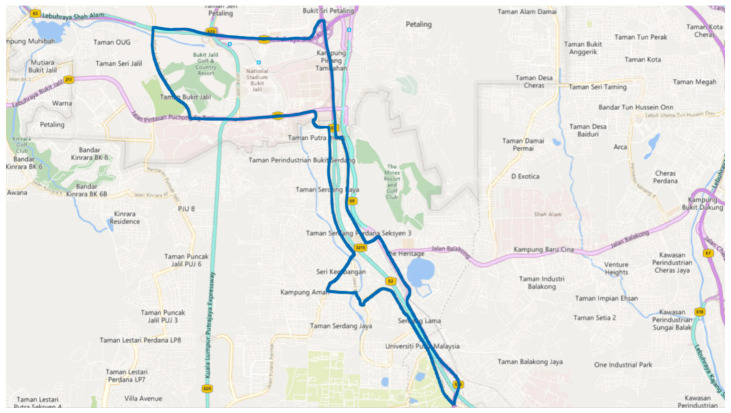
Selected test route.

Figure 12 depicts the experimental route, which was approximately 25 km long and included highways, intersections, roundabouts, 2-way lanes roads, 3-way lanes roads, and tunnels. The route was primarily urban and sub-urban and passed through two cities, Kula Lumpur and Serdang.

### 2.4. Data Collection Procedure

To ensure consistency, experiments were conducted throughout the week on clear sunny days from 9 a.m. to 12 p.m. Thus, external factors such as weather and visibility would not influence the data collection process and skew the future analysis. Temperature checks were performed, and participants were only permitted to drive if their readings were less than 37 °C (98.6 °F). Participants were also required to wear face masks and protective gloves during the experiments. In addition, only the participating driver was present inside the vehicle, and no instructions on how to drive were given. The driver’s movements were tracked through the GPS. The vehicle was thoroughly sanitized after each experiment. At most, only one experiment was carried out per day. A total of 21 trips were carried out during weekdays and 9 during weekends. The total duration of the experiments was 1148.85 min. The longest trip took 52.91 min, while the shortest took 30.23, with an average of 38.29 min per trip.

### 2.5. Data Processing

Collected raw data were filtered in three phases:

- Phase one (removal of unrelated data): irrelevant data recorded during experiments, such as engine load, engine oil, exhaust gas temperature, fuel pressure, and kilometers traveled per liter, were removed.
- Phase two (removal of extra data): when experiments begin, there is a brief period (between 1 and 5 s) during which the driver is not driving, but the DAS is recoding data. Moreover, after the experiment’s end, there is a brief period (between 1 and 10 s) during which the driver stops driving, but the DAS continues to record data. Such data were deleted in this phase.
- Phase three (removal of missing data): missing data, null values, blank values, and duplicated data.

### 2.6. Experts and Safety Criteria

With the assistance of the Road User Behavioural Change Research Center director at the Malaysian Institute of Road Safety Research (MIROS), speeding, close distancing, aggressive steering, harsh acceleration, and harsh deceleration were identified as the main factors that contribute to accidents in Malaysia.

The DAS recorded the three parameters, speed, distance, and steering, during experiments. The remaining two parameters, acceleration and deceleration, were derived and calculated mathematically from speed. The Malaysian highway code, traffic regulations, published articles, and extensive discussions with MIROS experts were used to determine when drivers are considered aggressive in relation to the aforementioned factors. It is a straightforward process in terms of speeding, as drivers must adhere to the speed limits of the designated route.

In terms of distancing, according to the Malaysian highway code, drivers should maintain a distance of at least 4 m with the vehicle ahead for every 10 mph (15 kmh). The acceleration and deceleration threshold limits were adopted from the article reference [47]. Regrading steering, the yaw axis change per second was calculated, and their z-scores were then used to determine safe/aggressive steering behavior. Z-scores range from −3 standard deviations (far left of the normal distribution curve) to +3 standard deviations (far right of the normal distribution curve). The Z score is denoted as z = (x − μ)/σ, where x is the change in the yaw axis per second; μ is the mean; and σ is the standard deviation. The criteria for determining safe/aggressive behavior in terms of speeding, distancing, acceleration, deceleration, and steering are shown in Table 3

### 2.7. Hypotheses

Data were collected from 30 participants of different genders, ages, and nationalities. Such diverse data can be analyzed to provide answers to the research questions posed in the introduction section. For instance, if there are no significant differences between male and female drivers, gender does not influence drivers in Malaysia. Furthermore, if there are significant differences between young and senior drivers, age has an impact on drivers in Malaysia. Therefore, several hypotheses were proposed:

**Hypothesis** **1** **(H1).** *Gender differences in driving are significant*.

**Hypothesis** **2** **(H2).** *There are significant differences in driving across age groups*.

**Hypothesis** **3** **(H3).** *Driving differs significantly between people of various cultural backgrounds*.

**Hypothesis** **4** **(H4).** *There are significant behavioral differences between people driving on weekdays and weekends*.

### 2.8. Independent Sample t-Test and ANOVA

In this study, drivers are categorized into two groups based on gender (males and females), two groups based on cultural background (local drivers and foreign drivers), three groups based on age (young, middle, and senior), and two groups based on driving day (weekday and weekend). An independent sample *t*-test was deployed in this study to find the differences among drivers in the two group categories. However, ANOVA was deployed to find the difference among drivers in the three age groups.

The Statistical Package for the Social Sciences (SPSS) was used to run the t-tests and ANOVA. According to Pallant [48], the researcher must examine Levene’s test for equality of variances in an independent sample t-test. If Levene’s sig value is greater than 0.05, then equal variances are assumed. However, if Levene’s sig value is less than 0.05, then equal variances are not assumed. The significant differences are confirmed if the t-test sig (2-tailed) is below 0.05. However, no differences are assumed if the value of the *t*-test sig (2-tailed) is above 0.05. All the analyses of the independent t-tests in this study were based on this rule. Moreover, post hoc tests were conducted to uncover specific differences between the three age groups when ANOVA tests showed significant differences.

## 3. Analysis Results

Aggressive events refer to the number of times the driver violated safety driving criteria listed in Table 3. The mean and aggressive events of the five driving parameters were used to compare the groups in the proposed hypotheses.

The *t*-test results revealed significant differences in average speed (sig 2-tailed = 0.001) and aggressive steering events (sig 2-tailed = 0.046) between male and female drivers, as shown in Table 4. The results showed that female drivers (mean = 48.20) drove significantly faster than male drivers (mean = 42.62). However, male drivers (mean = 141.80) performed more aggressive steering than female drivers (mean = 123.26).

Moreover, *t*-test results revealed significant differences between local and foreign drivers in terms of average steering (sig 2-tailed= 0.011) and aggressive steering events (sig 2-tailed = 0.008), as seen in Table 5. Local drivers steered much more frequently (mean = 9.66) and aggressively (mean = 144.60) than foreign drivers.

Moreover, *t*-test results revealed significant differences between driving on weekdays as opposed to weekends in terms of average speed (sig 2-tailed = 0.001), average decelerations (sig 2-tailed = 0.01), and aggressive steering events (sig 2-tailed = 0.34), as seen in Table 6. Drivers drove faster on weekends (mean = 49.72) than on weekdays (mean = 43.56). Drivers decelerated significantly more often on weekends (mean = −0.82) than during the week (mean = −0.76). Conversely, drivers steered more aggressively on weekdays (mean = 138.95) than on weekends (mean = 117.55).

ANOVA results showed significant differences between age groups in average speed (sig = 0.026) and average steering (sig = 0.030), as seen in Table 7. Post hoc tests were conducted to identify specific differences between the groups. The post hoc test, as shown in Table 8, revealed that there was a difference in average speed between drivers in the senior and middle groups. The sig value was 0.033, indicating that the differences between the two groups were statistically significant. Middle-aged drivers drove faster (mean = 47.49) than senior drivers (mean = 41.99). In addition, there were differences between drivers in the young group and drivers in the senior group in terms of average steering as the sig value was 0.024. Young drivers (mean = 9.79) steered more often than senior drivers (mean = 8.90).

Based on the findings of the analysis, it is safe to conclude that gender, cultural background, day of driving, and age significantly impact driving in Malaysia. As a result, H1, H2, H3, and H4 were accepted.

Though factors such as age, gender, driving day, and cultural background may appear to be independent, determining whether there is multicollinearity between these factors is critical because it undermines the statistical significance of an independent factor. Multicollinearity exists whenever an independent factor is highly correlated with other independent factors. As a result, correlation coefficient tests are utilized to check the significant relationships between the aforementioned factors and determine the strength and direction of the association. Correlation values range between −1 and +1. Those numbers indicate the strength of the correlation between two factors, while the sign indicates the relationship’s direction. For instance, −1 indicates a perfect negative correlation between factors, while +1 indicates a perfect positive correlation. However, 0 means no correlations whatsoever. The closer the number to +1 or −1, the stronger the magnitude of the relationship. According to the general guidelines, a correlation between two factors exceeding 0.90 indicates that the two factors are highly correlated [49,50]. This is also an indication that the factors are multicollinear. It is indicated that a correlation greater than 0.5 between two factors is considered strong, a correlation between 0.3 and 0.5 is considered moderate, and a correlation less than 0.3 and 0.1 is considered weak [51].

Table 9 displays the result of testing the correlation between factors. Significant correlations with a *p*-value less than 0.05 are labeled with a single star (*), while those with a *p*-value less than 0.01 are labeled with two stars (**). Results indicate a moderate negative correlation between age and gender with a coefficient of −0.49 and a *p*-value less than 0.01. Furthermore, results show a moderate positive correlation between gender and driving day with a coefficient of 0.36 and a *p*-value less than 0.05. There were no significant correlations found between the remaining factors. Given that the highest correlation coefficient was well below 0.9, the chances of multicollinearity between the factors are slim.

## 4. Discussion

This study’s results are comparable to previous publications. Researchers discovered that young drivers tailgated more aggressively than middle-aged and older drivers [8]. However, there were no differences in headways between young, middle, and senior drivers in this study. Researchers concluded that old drivers perform better than younger drivers in reference [7], which is relatively consistent with this study’s conclusions. Senior drivers had better steering performance than young drivers and were less hasty than middle-aged drivers. Results were also compatible with the findings from article references [11,52], which demonstrated that younger drivers were more likely to speed than older drivers.

Previous studies on gender were inconclusive and inconsistent, with some finding that male drivers were more likely to speed than female drivers [11]. Others found gender to be an insignificant contributing factor in speeding [53]. This study concluded, however, that gender plays a substantial role in driving, as female drivers drove significantly faster than males, while male drivers were more aggressive during steering maneuvers. In addition, the results were more consistent with the findings of the reference article [13], which showed that females and young drivers are faster than males and senior drivers.

According to previous research, drivers are 24% more likely to drive below the speed limit on weekends because they are not under pressure to get to work [13]. However, in this study, drivers drove faster on weekends than on weekdays. The authors believe that people drive faster on weekends in Malaysia because traffic tends to be less congested.

Researchers noted that Malaysian drivers required a higher danger threshold than UK drivers before identifying a hazardous situation [19]. However, their findings were limited in determining whether drivers from different cultures in Malaysia drive differently. This study revealed that cultural background influences driver behavior in Malaysia, as local drivers exhibited significantly more aggressive steering behavior than foreign drivers.

Correlation coefficient tests were used to confirm that there is no multicollinearity between factors, thereby ensuring the interpretation of the statistical results. Before issuing driving licenses, Malaysia’s Road Transport Department is advised to evaluate safe steering maneuvers for adolescent drivers. Policymakers should educate the public, particularly middle-aged drivers, about the importance of adhering to speed limits. Policymakers should also aim to raise public awareness on the importance of adhering to speed limits on weekends, even if traffic is light. The Ministry of Transport should provide driving instructors with tailored training courses that teach female drivers the importance of driving below the speed limit and male drivers the importance of lane-keeping behavior. Furthermore, local drivers were found to zigzag more frequently and more dangerously than foreign drivers. As a result, unique road signs should be placed to remind drivers not to switch lanes unless necessary.

## 5. Conclusions 

An efficient and cost-effective DAS was built in this study to collect driving data from naturalistic experiments in Malaysia. The majority of DAS sensors and equipment was hidden inside the vehicle to avoid influencing drivers’ behavior. The study collected over 750 km of continuous driving data from 30 drivers across two cities in Malaysia. First, safe and aggressive thresholds regarding driving parameters, such as speed, distance, steering, acceleration, and deceleration, were outlined. Then, aggressive events were calculated for each driver based on those thresholds. The study further explored the impact of social and cultural factors on driving, and four hypotheses were proposed. Those hypotheses assumed that drivers differed in terms of gender, age, cultural background, and driving day. The proposed hypotheses were tested using mean and aggressive events of each driving parameter. Drivers were categorized into two groups based on their gender (males and females), two groups based on their cultural background (local drivers and foreign drivers), three groups based on their age (young, middle, and senior), and two groups based on their driving day (weekday and weekend). Independent sample t-tests were deployed to find the differences among drivers in the two group categories. In addition, ANOVA was deployed to find differences among drivers in the three age groups. Results showed that female drivers drove faster than male drivers. Male drivers, on the other hand, steered more aggressively than their female counterparts. In addition, drivers in the middle age group drove faster than drivers in the senior age group, and drivers in the younger age group steered more frequently than drivers in the senior age group. Moreover, local drivers steered and changed lanes more frequently than foreign drivers. Furthermore, on weekends, drivers drove faster and decelerated more often. On weekdays, however, they steered rather aggressively. Based on the results, the proposed hypotheses were accepted. 

Based on the findings, the study outlined recommendations to various public sectors and policymakers in Malaysia, such as the Road Transport Department and the Ministry of Transport, to help reduce future accidents.

As far as the authors’ knowledge, this is the first naturalistic driving study in Malaysia. In the future, the authors intend to collect driving data from a larger sample size, improve the DAS accuracy by incorporating more sensors, and develop a deep-learning-based recognition system that can classify drivers based on their safe/aggressive behaviors. Thus, the DAS and the recognition system would help traffic police and insurance companies detect errant driving behaviors and improve traffic safety in Malaysia. 

## Figures and Tables

**Table 1:** Sensors, recoded parameters, and location.

Sensor	Sensor Location	Recorded Data
OBDII	Below the steering wheel	Speed
Lidar	Front of the vehicle	Long distances
Ultrasonic sensors	Front of the vehicle	Short distance
IMU	Inside the steering wheel	Steering
GPS	Vehicle trunk	Vehicle position

**Table 2:** Comparisons of the two lidar sensors (TF03 and Garmin).

Lidar Sensor	Recorded Distance Data	Accuracy Rate
Garmin	73%	82%
TF03	32%	45%

**Table 3:** Criteria for safe and aggressive behaviors.

Parameter	Criteria	Status
Speed	<speed limit	Safe
	>speed limit	Aggressive
Distance	>4 m for every 15 khm	Safe
	<4 m for every 15 khm	Aggressive
Acceleration	<3.5 m/s^2^	Safe
	>3.5 m/s^2^	Aggressive
Deceleration	>−5.5 m/s^2^	Safe
	<−5.5 m/s^2^	Aggressive
Steering	If z-score for the change in yaw axis per second is between 1σ and−1σ	Safe
	If z-score for the change in yaw axis per second is above 1σ or below −1σ	Aggressive

**Table 4:** Statistical differences in gender group.

Variables	Gender	N	Mean	Levene’s Test for Equality of Variances		*t*-Test for Equality of Means	
				F	Sig.	Sig. (2-Tailed)	Conclusions
Average speed	Male	15	42.62	0.192	0.664	0.001	Females drive faster than males.
	Female	15	48.20			0.001	
Steering aggressive events	Male	15	141.80	0.002	0.969	0.046	Males do more aggressive steering than females.
	Female	15	123.26			0.046	

**Table 5:** Statistical differences in cultural background group.

Variables	Nationality	N	Mean	Levene’s Test for Equality of Variances		*t*-Test for Equality of Means	
				F	Sig.	Sig. (2-Tailed)	Conclusions
Average steering	Local	15	9.66	4.767	0.038	0.010	Local drivers average significantly higher than foreign drivers
	Foreigner	15	8.96			0.011	
Steering aggressive events	Local	15	144.60	0.921	0.345	0.008	Local drivers do more aggressive steering than foreign drivers.
	Foreigner	15	120.46			0.008	

**Table 6:** Statistical differences in driving day group.

Variables	Day of Driving	*N*	Mean	Levene’s Test for Equality of Variances		*t*-Test for Equality of Means	
				F	Sig.	Sig. (2-Tailed)	Conclusions
Average speed	Weekday	21	43.56	0.199	0.659	0.001	Drivers drive faster on weekends than on weekdays.
	Weekend	9	49.72			0.001	
Average deceleration	Weekday	21	−0.76	0.273	0.605	0.010	Drivers decelerate more often on weekends than on weekdays.
	Weekend	9	−0.82			0.031	
Steering aggressive events	Weekday	21	138.95	0.105	0.748	0.034	Drivers steer more aggressively on weekdays than on weekends.
	Weekend	9	117.55			0.037	

**Table 7:** Statistical differences in age group.

Variable	Age	N	Mean	F	Sig.	Conclusions
Average speed	Young	10	46.76	4.193	0.026	There are differences between age groups with relation to average speed
	Middle	10	47.49			
	Senior	10	41.99			
	Total	30	45.41			
Steering aggressive events	Young	10	9.79	4.017	0.030	There are differences between age groups with relation to average steering
	Middle	10	9.25			
	Senior	10	8.90			
	Total	30	9.31			

**Table 8:** Post hoc results to highlight differences in age group.

Dependent Variable	(I) Age	(J) Age	Sig.	Conclusions
Average speed	Middle	Young	0.933	Middle drivers drive faster than old drivers.
		Old	0.033	
	Senior	Young	0.071	Old drivers drive slower than young drivers.
		Middle	0.033	
Average steering	Young	Middle	0.221	Young drivers steer significantly more than old drivers.
		Old	0.024	
	Senior	Young	0.024	Old drivers steer significantly less than young drivers.
		Middle	0.519	

**Table 9:** Correlation matrix.

Variable	Gender	Cultural Background	Age	Driving Day
Gender	1			
Cultural Background	0.20	1		
Age	−0.49 **	−0.08	1	
Driving Day	0.36 *	0.07	−0.08	1

* Correlation is significant at the 0.05 level (2-tailed). ** Correlation is significant at the 0.01 level (2-tailed).

## Data Availability

Provided by the authors upon request.
